# A Leaf-Vein-Inspired Composite Flow Channel for Enhanced Mass Transport and Performance in Proton Exchange Membrane Fuel Cells

**DOI:** 10.3390/membranes16090287

**Published:** 2026-08-28

**Authors:** Tingjie Ba, Hongyi Zeng, Wanjun Wu, Dong Jiao, Yongyuan Huang

**Affiliations:** 1 Yunnan Power Grid Co., Ltd., Kunming 650000, China; 2 Faculty of Traffic Engineering, Kunming University of Science and Technology, Kunming 650000, China; 3 Diqing Power Supply Bureau, Yunnan Power Grid Co., Ltd., Diqing 674400, China

**Keywords:** proton exchange membrane fuel cell (PEMFC), leaf vein–serpentine composite flow field, water thermal management, multiphysics simulation

## Abstract

A leaf-vein–serpentine composite flow field (L-SFF) is proposed to improve reactant distribution, water management, and electrochemical performance of proton exchange membrane fuel cells (PEMFCs). The L-SFF consists of a main trunk and multilevel branches inspired by coconut palm leaf veins, with a branching angle of 45° and a branch width of 2 mm. Three-dimensional multiphysics models of the traditional serpentine flow field (SPFF), cathode leaf-vein–serpentine flow field (S-LFF), and anode leaf–vein–serpentine flow field (L-SFF) were developed using COMSOL Multiphysics 6.1 to investigate current density, membrane water content, temperature distribution, and power output. Under 0.6 V, 353 K, and 1 bar conditions, the L-SFF achieves a maximum power density of 0.538 W·cm^−2^, approximately 2.1% higher than SPFF, with an average anode current density of 7092.0 A·m^−2^ and a current-density uniformity index of 0.135. The L-SFF also exhibits improved membrane water distribution, with an average water mole fraction of 0.513, and maintains an average membrane temperature of 356.61 K. Furthermore, the effect of GDL porosity on performance was evaluated. When the GDL porosity decreases from 0.8 to 0.2, the maximum power density decreases from 0.534 to 0.474 W·cm^−2^ for SPFF (11.2%) and from 0.547 to 0.489 W·cm^−2^ for L-SFF (10.6%), indicating better tolerance to variations in porous transport properties. The results demonstrate that the L-SFF structure enhances reactant transport, water management, and performance stability, providing an effective strategy for PEMFC flow-field optimization.

## 1. Introduction

Proton Exchange Membrane Fuel Cells (PEMFCs), as a pivotal component of clean energy technology, exhibit high energy conversion efficiency, low operating temperature, zero carbon emissions, and rapid start-up characteristics. Consequently, they demonstrate broad application prospects in transportation, distributed power generation, and portable energy systems [1,2,3]. Particularly in the context of global energy transition and carbon emission constraints, PEMFCs serve as a crucial pillar for renewable energy and hydrogen-based systems, holding considerable market potential and engineering significance.

Despite significant progress in PEMFC technology, their operational performance remains constrained by multiple factors, among which internal flow field design plays a central role in governing reactant gas distribution, water management, and thermal regulation [4]. Improper flow channel layouts can cause uneven reactant distribution, local water accumulation, and thermal non-uniformity, increasing internal pressure losses and potentially triggering performance degradation or localized failure [5,6]. These limitations can reduce cell output power and energy efficiency and compromise operational stability. Therefore, optimizing flow field structures, particularly those capable of simultaneously ensuring uniform gas distribution, efficient water removal, and effective heat transfer, remains critical for improving overall cell performance and durability.

Conventional PEMFC flow fields primarily include parallel, serpentine, and interdigitated geometries. Parallel channels offer simple manufacturing and low pressure drop but, due to limited branching and guiding mechanisms, often result in uneven reactant distribution across the electrode, leading to oxygen starvation and reduced local reaction rates [7]. Serpentine channels, with continuous long bends, provide higher pressure drive favorable for water removal and stable single-cell performance. However, in large-area cells or high-current-density operation, they suffer from localized high pressure, increased energy consumption, and uneven two-phase flow [8]. Interdigitated channels improve reactant transport through forced convection, enhancing mass transfer efficiency but at the expense of high manufacturing complexity and stringent material and sealing requirements. Bunyan et al. [9] compared parallel and serpentine channels, confirming that parallel channels are easier to fabricate but exhibit uneven reactant distribution. Ahmed et al. [10] reviewed serpentine channel studies, highlighting their effective water removal but noting high local pressures in large-area cells. Liu et al. [11] analyzed interdigitated channels via CFD and experiments, revealing improved mass transport but higher manufacturing complexity. Wang et al. [12] pointed out that serpentine channels generate high local pressures, while parallel channels in large-area cells suffer from uneven gas distribution, limiting performance improvements. As fuel cells trend toward high power density and large-area designs, these traditional configurations reveal increasing shortcomings in gas uniformity, water thermal management, and pressure loss control, restricting PEMFC performance enhancement and large-scale deployment.

To overcome these limitations, researchers have drawn inspiration from natural biological structures in recent years. Xuan et al. [13] proposed 3D bio-inspired cathode flow fields that improved gas distribution and water management. Huang et al. [14] applied leaf-vein structures to PEMFC channels, demonstrating enhanced gas uniformity and anti-flooding capability. Iranzo et al. [15] investigated lung-like and dendritic biomimetic channels, achieving multi-scale mass and heat transport. Natural transport systems-such as leaf veins, alveoli, blood vessels, and dendritic networks-exhibit high efficiency, low resistance, and multi-scale coordination in material and energy transport [16]. Translating these geometric characteristics into PEMFC flow field design enables simultaneous macro- and micro-scale optimization of reactant distribution, water removal, and thermal conduction. Gao et al. [17] constructed leaf-vein-inspired channels and validated improved reactant distribution via CFD. Ala’a et al. [18] developed multi-scale biomimetic networks, showing superior thermal and local water management compared to traditional designs. Jian et al. [19] experimentally confirmed that biomimetic channels outperform conventional ones in water thermal management and pressure loss. Leaf-vein flow fields employ primary and secondary branching to distribute gases uniformly across active areas, while lung-like or dendritic channels achieve efficient multi-scale mass and heat transport, mitigating local flooding and overheating [20,21,22]. These biomimetic designs have been widely validated in recent studies, demonstrating significant advantages in reducing local pressure losses, enhancing water thermal management, and increasing overall cell power density, offering a novel design paradigm for conventional flow fields.

In recent years, biomimetic PEMFC flow field research has rapidly advanced. Xia et al. [23] optimized leaf-vein flow fields, significantly reducing pressure drop and improving reactant distribution. Xiao et al. [24] conducted multi-objective optimization on leaf-vein geometries to enhance water removal efficiency. Han et al. [25] combined experiments and numerical methods to investigate biomimetic design impacts on water management. Nuttapol et al. [26] proposed serpentine-interdigitated composite flow fields to balance gas distribution and water removal. Kim et al. [27] combined serpentine and parallel channels to improve mass transfer uniformity. Dong et al. [28] used CFD for multi-objective optimization of complex biomimetic flow parameters. Ze et al. [29] applied genetic algorithms to optimize flow channel geometry, achieving high performance with low pressure loss.

Although various biomimetic flow fields, including leaf-vein, dendritic, fractal, and interdigitated structures, have been developed to improve reactant transport and water management, each design has certain limitations. Leaf-vein and dendritic structures provide efficient multi-path transport but may face challenges in water removal, while fractal and interdigitated structures often involve complex optimization, increased pressure loss, or fabrication difficulties. Therefore, developing a simple and effective composite flow-field structure that simultaneously achieves uniform reactant distribution, efficient water transport, acceptable pressure loss, and practical manufacturability remains an important research objective. Furthermore, most previous studies have mainly focused on optimizing flow-field geometries, whereas the coupling effect between flow-field structures and GDL porous transport properties has not been sufficiently investigated.

In addition, gas diffusion layers (GDLs) play a critical role in PEMFCs by facilitating water and gas transport, heat conduction, and electron transfer, while providing mechanical support to the catalyst layer and membrane during assembly [30,31,32,33,34]. The GDL’s structural characteristics directly influence electrochemical reaction progress and overall cell performance [35]. However, previous studies on biomimetic flow fields and GDL structures have generally been conducted independently, and the synergistic influence of flow-field architecture and GDL transport properties on PEMFC performance remains insufficiently explored. Therefore, coupling the optimization of flow-field structures with the regulation of GDL transport characteristics represents an important direction for improving PEMFC performance and operational stability. Wei et al. [36] employed stochastic reconstruction methods to compare and analyze the pore structures of different GDL types and, using pore-scale modeling, computed anisotropic effective transport properties, revealing the influence of pore distribution on gas diffusion and liquid water management. Shang et al. [37] studied the effect of planar pore gradients on gas–liquid transport and cell performance, finding that moderate gradients optimize water pathways and increase current density. Sun et al. [38] analyzed water transport behavior from a pore distribution perspective, showing that specific porosity distributions significantly improve liquid water removal efficiency and overall cell performance.

Based on this background, the present study proposes a leaf-vein–serpentine biomimetic composite flow field to improve reactant distribution, water management, and heat transfer, while further investigating the influence of GDL porosity on transport characteristics and cell performance. This design combines the multi-branch transport characteristics of leaf-vein structures with the water–thermal management capability of serpentine channels, and evaluates the coupling effects between flow-field architecture and GDL transport properties. The proposed strategy aims to enhance reactant distribution and water management while improving PEMFC performance and operational stability.

Overall, the proposed leaf-vein–serpentine biomimetic composite flow field provides a novel design strategy by integrating biomimetic channel architecture with porous-media transport optimization. This work highlights the importance of coupling flow-field design and GDL transport characteristics for achieving improved reactant distribution, water–thermal management, and stable PEMFC operation.

## 2. Materials and Methods

### 2.1. Geometric Model

Traditional fuel cell flow fields often suffer from uniform channel arrangements and lack of optimized layouts, which can lead to uneven gas distribution and inefficient heat and water transport. Inspired by the natural hydrophobic structure of coconut palm leaves, this study proposes a leaf-vein-inspired biomimetic flow field. The design coordinates reactant gas and liquid water transport through a primary trunk with multi-level branching, achieving a synergistic combination of macro-scale uniformity and micro-scale water thermal management. The main trunk guides gas flow toward each branch, while the branches accelerate liquid water removal, effectively reducing local pressure drop and water accumulation. To evaluate the design performance, single-cell models of a conventional serpentine flow field (SPFF), a cathode leaf-vein–serpentine composite flow field (S-LFF), and an anode leaf-vein–serpentine composite flow field (L-SFF) were established. Three-dimensional multiphysics numerical simulations were performed using COMSOL Multiphysics 6.1. Furthermore, for the leaf-vein composite flow field, the inlet and outlet positions were optimized to coordinate reactant supply and water removal paths, thereby maximizing the overall water thermal management performance of the cell.

**Figure 1:**
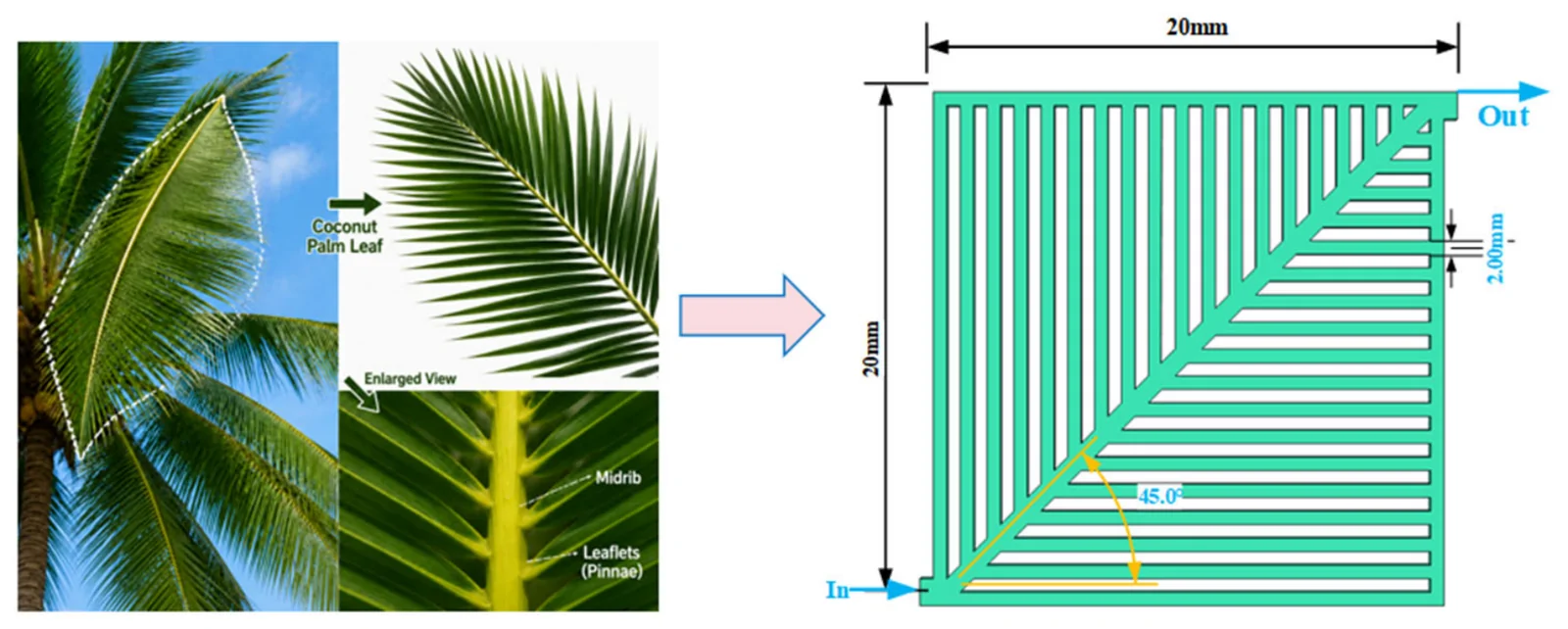
Structure of the PEMFC geometric model.

Figure 1 illustrates the leaf-vein–serpentine composite flow field (L-SFF) inspired by the structure of a coconut palm leaf. Upon entering the cell, the reactant gases are guided along the main trunk channel and distributed to the multiple branches, where they participate in electrochemical reactions within the catalyst layer (CL). Meanwhile, the gas diffusion layer (GDL) assists in gas transport and facilitates the removal of liquid water. Unreacted gases and generated water vapor are discharged through the outlet. The L-SFF flow field features an active area of 20 mm × 20 mm, branch widths of 2 mm, and a 45° branching angle, optimized to achieve uniform gas distribution and rapid liquid water evacuation while maintaining low pressure drop and manufacturability. Table 1 presents the geometric parameters of the fuel cell used in the simulations, and Table 2 summarizes the operating conditions and physical properties applied during the calculations.

### 2.2. Governing Equations

The governing equations used in the simulation process by the software are as follows:

The basic fluid mechanics equations.

Mass conservation equation [39]:

(1) $$\nabla \cdot (\rho U)=S_{m}$$

where ρ is the density, U is the velocity, and S_m_ is the mass source terms.

Energy conservation equation:

Assuming that the liquid flows in the flow field without considering the viscosity of the liquid and that the liquid flows in a steady, slow manner in the flow field, the energy conservation equation [40] can be obtained:

(2) $$\frac{\partial \left(\varepsilon \rho C_{p}T\right)}{\partial t}+\nabla T\cdot \rho C_{P}U-\nabla \cdot \left(k^{\mathrm{eff}}\nabla T\right)=S_{d}$$

where C_p_ is the specific pressure heat capacity, $k^{\mathrm{eff}}$ represents the effective thermal conductivity, T is the temperature, and Sd is the energy source term, including electro-chemical reaction heat, ohmic heat, water evaporation–condensation heat, and water adsorption–dissolution heat.

Momentum conservation equation:

Assuming all liquids in the PEMFC are Newtonian fluids, we analyze the normal and shear stresses applied to the liquid surface. By applying the Navier–Stokes viscosity theorem, it compares the motion changes in liquid components in different regions with the influence of external forces. This yields the momentum conservation equation as follows [41]:

(3) $$\frac{1}{\varepsilon^{2}}+\nabla \cdot (\rho UU)+\nabla p+\frac{1}{\varepsilon }\nabla \cdot (\mu \nabla U)=S_{v}$$

where *p* is the pressure, *μ* is the viscosity, and *S*_v_ is the momentum source terms.

Current conservation equation:

The current conservation equation is as follows:

(4) $$\nabla \cdot \left(\sigma_{\mathrm{sol}\nabla }\phi_{\mathrm{sol}}\right)+R_{\mathrm{sol}}=0$$

(5) $$\nabla \cdot \left(\sigma_{\mathrm{men}\nabla }\phi_{\mathrm{men}}\right)+R_{\mathrm{men}}=0$$

where $\sigma_{s}$ is the conductivity, ϕ is the phase potential, and R represents the exchange current density between the anode and cathode of the cell; the subscripts sol and men denote the solid phase and membrane phase, respectively. In other regions, $R_{\mathrm{sol}}=R_{\mathrm{men}}=0$ within the catalyst layer, the volume exchange current density is non-zero.

Electrochemical equations:

The relationship between activation overpotential and volume exchange current density can be determined by solving the Butler–Volmer equation [42]:

(6) $$R_{a}=(\xi_{a}j_{a}^{ref}){\left(\frac{C_{H_{2}}}{C_{H_{2}}^{ref}}\right)}^{\gamma_{a}}(e^{\frac{\alpha_{a}F}{RT}\eta_{a}}-e^{-\frac{\alpha_{c}F}{RT}\eta_{a}})$$

(7) $$R_{c}=(\xi_{c}j_{c}^{ref}){\left(\frac{C_{O_{2}}}{C_{O_{2}}^{ref}}\right)}^{\gamma_{c}}(-e^{\frac{\alpha_{a}F}{RT}\eta_{c}}+e^{-\frac{\alpha_{c}F}{RT}\eta_{c}})$$

where $j_{a}^{ref}$ is the anode current density, $j_{c}^{ref}$ represents the cathode current density, is the Catalyst layer specific surface area, ξ is the reference molar concentration of hydrogen gas, $C_{O_{2}}^{ref}$ represents the reference molar concentration of oxygen gas, $C_{H_{2}}$ is the local molar concentration of hydrogen gas, $C_{O_{2}}$ is the local molar concentration of oxygen gas, $\gamma^{c}$ is the cathode reference concentration exponent, $\alpha^{c}$ is the charge transfer coefficient, *T* is the component temperature, η is the activation over potential, and *R* is the ideal gas constant. The formula for calculating the reference exchange current density $j_{a,c}^{ref}$ is as follows [42]:

(8) $$j_{a}^{ref}=10^{9}\cdot e^{\left[-1400\left(\frac{1}{T}-\frac{1}{353.15}\right)\right]}$$

(9) $$j_{c}^{ref}=10^{4}\cdot e^{\left[-7900\left(\frac{1}{T}-\frac{1}{353.15}\right)\right]}$$

The activation overpotential can be determined using the following calculation formula:

(10) $$\eta_{\mathrm{ax}}=\phi_{s}-\phi_{m}$$

(11) $$\eta_{\mathrm{cat}}=\phi_{s}-\phi_{m}-V_{\mathrm{oc}}$$

where *V*_oc_ is the open-circuit voltage.

Water transport equation:

Water plays a critical role in maintaining membrane hydration and electrolyte conductivity. Excessive water accumulation, however, may impede reactant transport through porous media and deteriorate cell performance. In PEMFCs, water transport is governed by electro-osmotic drag, pressure-driven flow, and concentration-driven diffusion.

(1) Electromigration water flux of the PEMFC:

(12) $$N_{w,y,e}=n_{d}\frac{1}{F}$$

where $n_{d}$ is the electromigration coefficient, and it depends on the water content of the membrane. The electromigration coefficient is $n_{d}=\frac{2.5\lambda }{22}$.

The relationship between λ (water content) and α (water saturation) is:

(13) $$\left\{\begin{array}{c} \lambda =0.043\ldots \ldots \alpha <0\\ \lambda =0.043+17.81\alpha -39.85\alpha^{2}+36.0\alpha^{3}\ldots \ldots 0\leq \alpha <1\\ \lambda =14.0+1.4(\alpha -1)\ldots \ldots 1\leq \alpha \leq 3\\ \lambda =16.8\ldots \ldots \alpha >3 \end{array}\right.$$

where $\alpha =\frac{p_{wv}}{p_{sat}(T)}+2s$, $P_{w}$ is the water vapor pressure, $p_{sat}(T)$ is the saturation pressure at temperature T, and *S* is the liquid water saturation within the cell.

At different temperatures, the saturation vapor pressure can be calculated using the following formula:

(14) $$P_{sat}(T)=101325\times 10^{\left(-2.1794+0.02953T+9.18337\times 10^{-3}T^{2}+1.4454\times 10^{-7}T^{3}\right)}$$

The proton conductivity of the membrane is:

(15) $$\sigma_{m}=(0.514\lambda -0.326)e^{1268\left(\frac{1}{303}-\frac{1}{T}\right)}$$

(2) Pressure-driven water flux of the PEMFC:

Pressure-driven migration describes water transport induced by a pressure difference across the membrane. Since the anode and cathode operating pressures are both maintained at 1 atm in this study, no pressure gradient exists across the membrane, and the pressure-driven water flux is therefore assumed to be negligible.

(3) Concentration difference diffusion water flux of the PEMFC:

(16) $$N_{m}=-D_{m}\frac{\partial_{c}}{\partial y}$$

where $D_{m}=D_{\lambda }\cdot e^{2416\left(\frac{1}{303}\frac{1}{T}\right)}$ is the diffusion coefficient of water in the PEM:

(17) $$\left\{\begin{array}{l} D_{\lambda }=10^{-10}\ldots \lambda <2\\ D_{\lambda }=10^{-10}\times \left[1+2\left(\lambda -2\right)\right]\ldots 2\leq \lambda \leq 3\\ D_{\lambda }=10^{-10}\times \left[3-1.67\left(\lambda -3\right)\right]\ldots 3<\lambda <4.5\\ D_{\lambda }=1.25\times 10^{-10}\ldots \lambda \geq 4.5 \end{array}\right.$$

Component Conservation Equation:

In electrochemical reactions, the reactants obey the law of mass conservation; the reaction mechanism has been experimentally verified. The mass flow rate of each component is influenced by both convective and diffusive processes; its conservation equation is as follows:

(18) $$\frac{\partial \left(\rho \varepsilon c_{i}\right)}{\partial t}+\nabla \cdot \left(\rho \varepsilon c_{i}\vec{v}\right)=\left(\rho D_{i}^{eff}\nabla c_{i}\right)+S_{i}$$

where *C_i_* denotes the concentration of species i, $D_{i}^{eff}$ represents the effective diffusion coefficient of species i, and *S_i_* is the corresponding species source term. The effective diffusion coefficient is calculated as follows:

(19) $$D_{i}^{eff}=\varepsilon^{1.5}(1-S{)}^{r_{i}}D_{i}^{0}{\left(\frac{P_{0}}{P}\right)}^{y_{p}}{\left(\frac{T}{T_{0}}\right)}^{y_{r}}$$

where *S* denotes the water saturation (kg/m^3^); $D_{i}^{0}$ is the diffusion coefficient of species i at the reference pressure P_0_ and temperature T_0_ (m^2^/s); rs represents the pore-blocking coefficient of the porous medium; and γ_p_ and γ_T_ are the pressure and temperature exponents, respectively.

In the catalytic layer, the source term for each component is zero; however, the source terms for hydrogen, oxygen, and water in the catalytic layer are

(20) $$S_{H_{2}}=-\frac{1}{2F}M_{H_{2}}R_{a}$$

(21) $$S_{O_{2}}=-\frac{1}{4F}M_{O_{2}}R_{c}$$

(22) $$S_{H_{2}O}=-\frac{1}{2F}M_{H_{2}O}R_{c}$$

The permeability of the GDL is also adjusted according to the porosity variation using the Carman–Kozeny relationship:

(23) $$K=K_{0}\frac{\varepsilon^{3}}{{\left(1-\varepsilon \right)}^{2}}$$

where *K* is the permeability under different porosity conditions, and *K*_0_ is the reference permeability. This relationship describes the influence of pore structure variation on convective transport resistance in the porous layer.

Heat transfer equation:

The heat transfer process in PEMFC mainly includes three parts: heat transfer in porous media, heat transfer in fluids, and heat transfer in solids [43]:

(1) Heat transfer equation in porous media:
  (24) $${\left(\rho C_{p}\right)}_{\mathrm{eff}}\frac{\partial_{T}}{\partial_{t}}+\rho_{f}C_{\mathrm{pf}}\mu \cdot \nabla T+\nabla \cdot q=Q$$
(2) Fluid heat transfer equation:
  (25) $$\rho C_{p}\left(\frac{\partial_{T}}{\partial_{t}}+\mu_{t}\cdot \nabla T\right)+\nabla \cdot \left(q+q_{r}\right)=T\left(\frac{\partial_{p}}{\partial_{t}}+\mu_{t}\cdot \nabla p\right)+\tau \cdot \nabla \mu +Q$$
  where τ is the viscous stress tensor.
(3) Solid heat transfer equation:
  (26) $$\rho C_{p}\left(\frac{\partial_{T}}{\partial_{t}}+\mu_{t}\cdot \nabla |T\right)+\nabla \cdot \left(q+q_{r}\right)=-\alpha T\frac{\mathrm{ds}}{\mathrm{dt}}+Q$$
  where $\mu_{t}$ is the translational velocity vector, *C*_p_ is the isochoric specific heat capacity, *q* is the thermal conductivity flux, α is the thermal expansion coefficient, *Q* is the heat source term, $q_{r}$ is the radiative heat flux.

To ensure the reliability of the simulation results, the following assumptions and simplifications are adopted during the solution of the governing equations:

(1) The PEMFC operates under steady-state conditions.
(2) All gases are treated as ideal and incompressible laminar flows.
(3) The GDL, CL, and membrane are modeled as homogeneous and isotropic porous media.
(4) The effects of gravity are neglected.

### 2.3. Boundary Conditions

The boundary conditions involve the interface conditions at the inlet and outlet of the flow channel, as well as the upper and lower surfaces of the PEMFC.

(1) Solid wall: no shear slip condition for the cathode and anode sides.
(2) The outlet of the channel on both the cathode and anode sides of the PEMFC adopts a pressure outlet, the size of which is atmospheric pressure under standard conditions.
(3) From the mass balance requirement of the PEMFC, it can be obtained that the sum of input masses must be equal to the sum of output masses, so the mass flow inlet is adopted.
(4) The electric and ionic fluxes at each boundary of the cell surface are zero, and the electrical conductivity of the solid and the ionic conductivity of the PEM are set to constants.
(5) The potential on the anode side is 0 V, and the electric potential on the cathode side is determined by the cell voltage.

### 2.4. Simulation Model Validation

**Figure 2:**
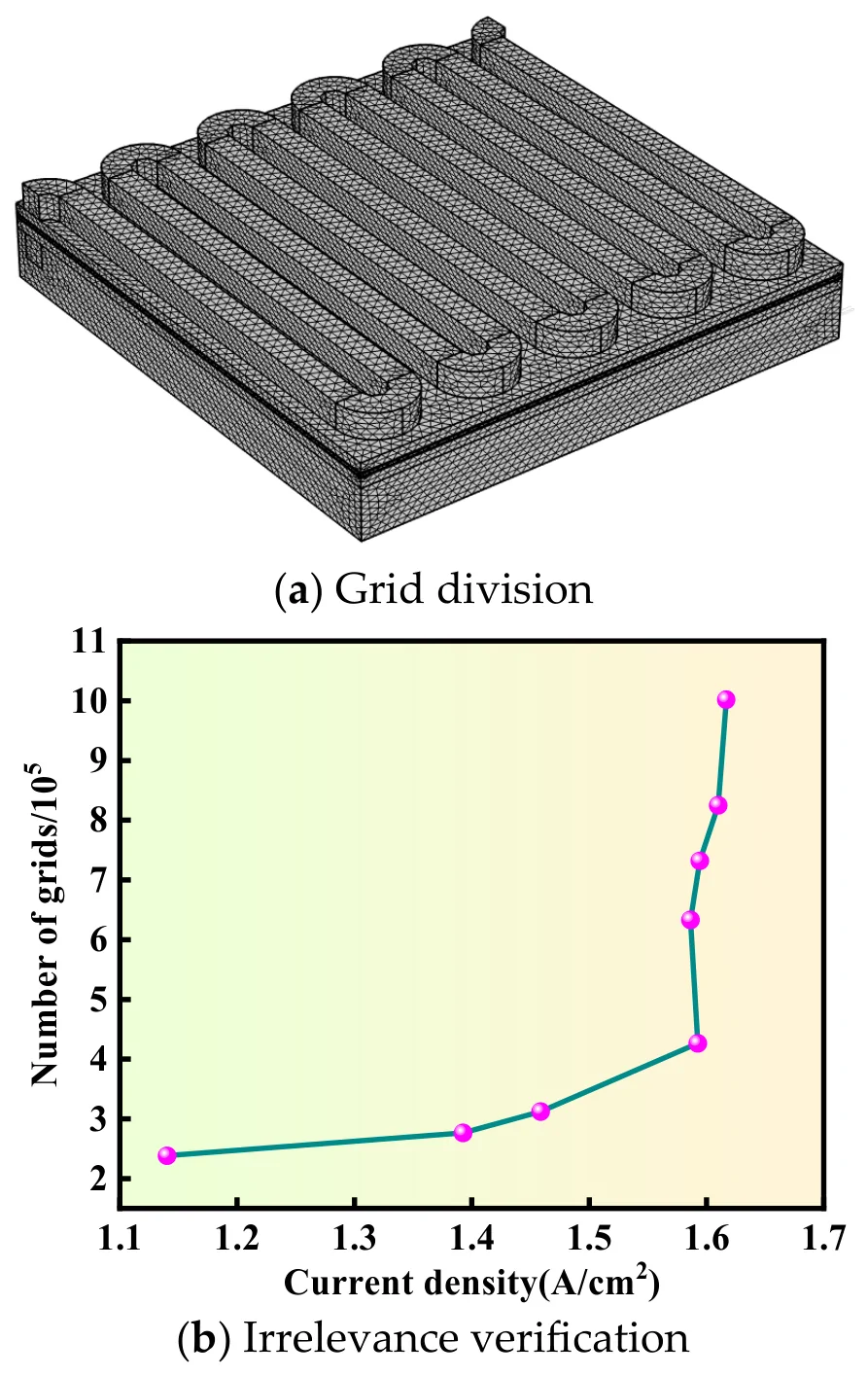
Grid division and grid-independence verification.

Before performing the numerical simulations, a grid independence study was conducted on the initial serpentine flow field (SPFF) PEMFC model to ensure the accuracy and reliability of the computational results. Current density was selected as the key indicator for evaluating cell performance. Figure 2 illustrates the effect of different mesh densities on current density under an output voltage of 0.6 V. The analysis shows that when the total number of mesh elements reaches approximately 400,000, the current density stabilizes, and further increases in mesh density have negligible impact on the results. Considering both computational accuracy and efficiency, a mesh of approximately 630,000 elements was selected as the standard computational grid for this study.

**Figure 3:**
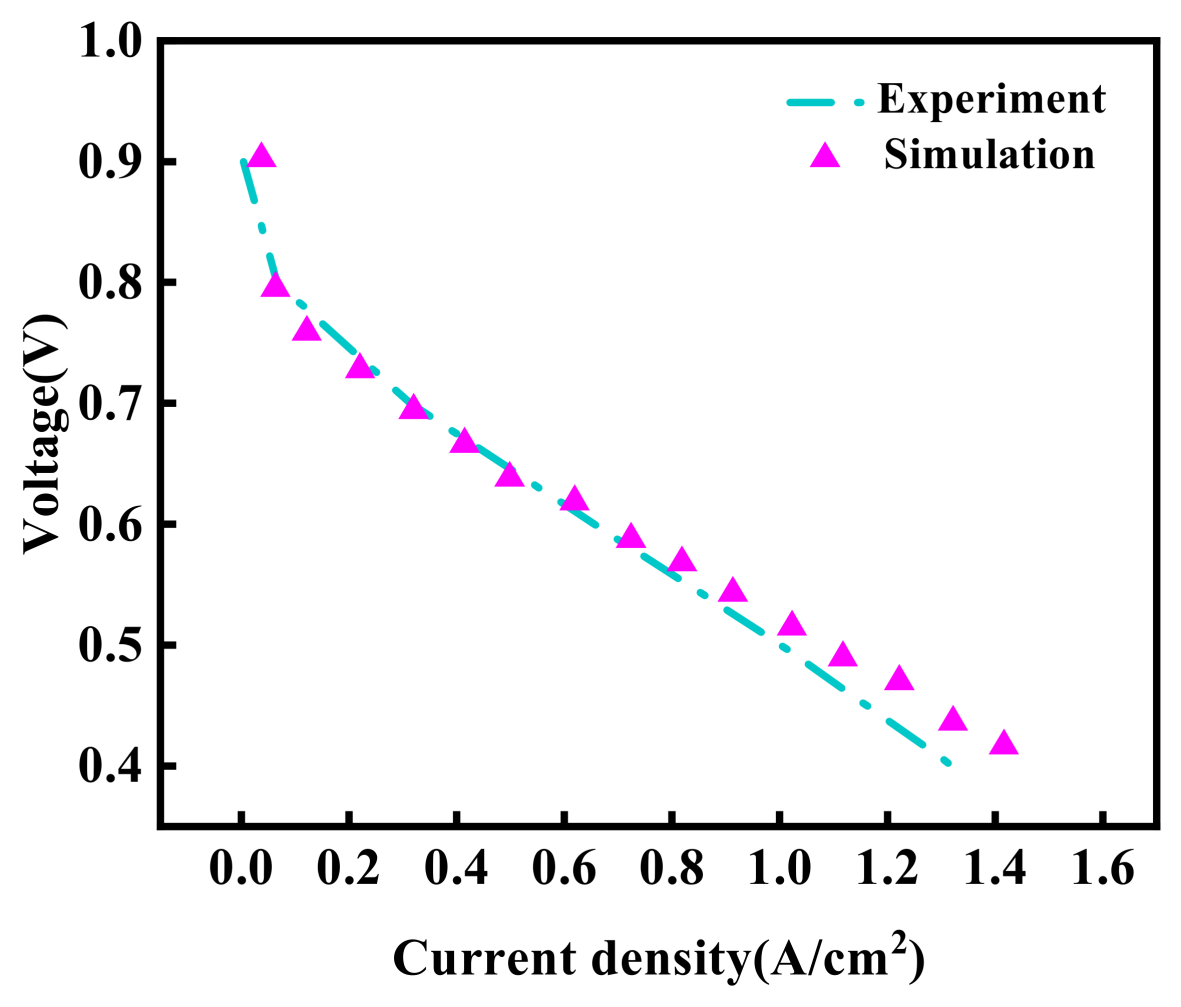
Model validation comparison.

To validate the reliability of the model, the simulation results were compared with existing experimental data. The results are shown in the Figure 3. Under operating conditions of 353 K, rectangular channel cross-section, and 1 bar pressure, the simulated polarization curve shows good agreement with the experimental data reported by Shang et al. [44], with deviations within 5%. These results demonstrate that the established SPFF model can reasonably predict the electrochemical behavior of PEMFCs under steady-state operating conditions. Therefore, the validated model was further applied to investigate the transport characteristics and performance variations in the proposed leaf-vein–serpentine composite flow field. Experimental validation of the proposed flow field remains necessary in future work.

## 3. Results and Discussion

To enhance gas uniformity and water thermal management in PEMFCs, this study proposes a leaf-vein–serpentine composite flow field (L-SFF) design. The design integrates the uniform transport characteristics of a multi-branch leaf-vein network with the long-channel water thermal management capability of serpentine channels, forming a multi-scale network of main trunks and branches. The main trunk channels are aligned along the flow direction to rapidly deliver reactant gases to the branches, while the branch channels extend from the trunk at a 45° angle with a width of 2 mm to ensure uniform gas distribution and efficient liquid water removal. Meanwhile, the rib width, channel depth, and active area between the trunk and branches are optimized according to cell design requirements to balance pressure drop and manufacturability.

For different configurations, polarization curves, power density curves, current density distributions, and membrane water content distributions were systematically compared. The combined influence of flow field structure and GDL porosity on PEMFC performance was comprehensively evaluated, providing insights for optimization from both flow field construction and porosity design perspectives.

To examine the specific effects of the leaf-vein flow field on PEMFC performance, the leaf-vein channels were placed separately at the cathode and anode, and a three-dimensional PEMFC model was constructed for multi-physics numerical simulation. Results indicate that the composite flow field achieves uniform reactant gas distribution at the macroscopic scale while efficiently removing liquid water at the microscopic scale, significantly mitigating local water accumulation and thermal hotspots. Analysis of polarization curves, power density, current density, and membrane water content demonstrates that the L-SFF flow field markedly enhances overall cell performance, providing a solid theoretical and quantitative foundation for subsequent composite flow field optimization and practical PEMFC applications. Detailed analysis and discussion of the results are presented as follows:

### 3.1. Performance Analysis of PEMFC Under Different Combined Flow Channel Configurations

#### 3.1.1. Polarization Curve and Power Density Curve

**Figure 4:**
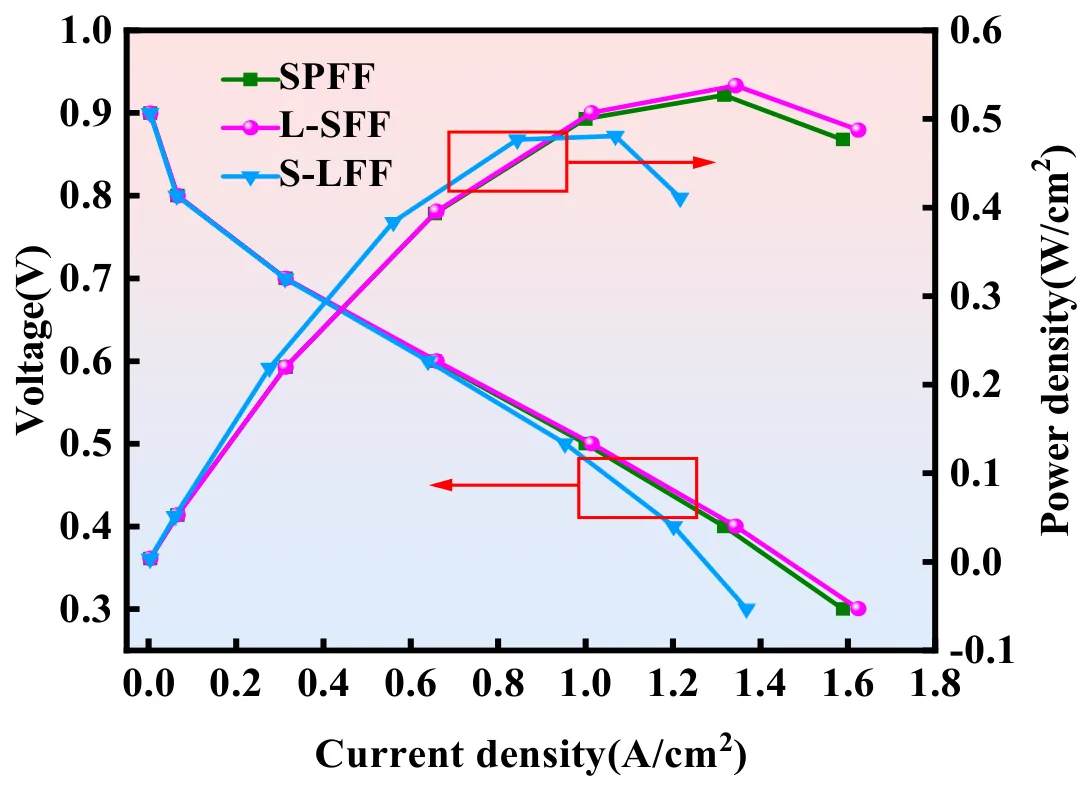
Current density and power density curves under different flow channel configurations.

To investigate the impact of leaf-vein flow field placement on fuel cell output performance, this study analyzed and compared configurations with the leaf-vein channels positioned at the anode and the cathode. The polarization and power density curves for the three flow field configurations are shown in Figure 4. As illustrated, positioning the leaf-vein flow field at the anode (L-SFF) significantly enhances fuel cell power density, reaching a maximum of 0.538 W/cm^2^, representing a 2.1% improvement compared with the baseline SPFF model. In contrast, placing the leaf-vein channels at the cathode results in lower performance, with a maximum power density of only 0.427 W/cm^2^, which is markedly below the baseline.

This performance difference is mainly attributed to the geometry-dependent water management characteristics of the serpentine and leaf-vein flow channels. The serpentine channels provide stronger pressure-driven drainage pathways, facilitating liquid water removal, whereas the leaf-vein channels focus on improving reactant distribution through multi-branch transport pathways. The different flow structures lead to variations in local water accumulation and reactant accessibility, thereby affecting electrochemical reaction efficiency. Excess water accumulation can degrade the durability of the GDL, CL, and PEM, increasing fuel cell costs and constraining industrial application. However, the leaf-vein flow field provides superior gas transport; when positioned at the anode, hydrogen is distributed more uniformly throughout the gas diffusion layer, enhancing reaction efficiency and overall fuel cell performance. Considering both output performance and economic factors, the L-SFF configuration is identified as the optimal flow field design for high-performance PEMFCs.

#### 3.1.2. Analysis of the Distribution of Water Molar Fraction at the Cathode

As discussed in the previous section, excessive water accumulation can negatively affect PEMFC performance. Therefore, analyzing the membrane water distribution under different flow field configurations is important for evaluating water management characteristics. In this section, the water content distribution within the proton exchange membrane (PEM) is investigated under various flow field geometries. The membrane water content reflects the hydration state of the PEM, where insufficient hydration may increase proton transport resistance, while excessive water accumulation may affect reactant transport and electrochemical activity. Moreover, the membrane water distribution provides insight into the overall water management behavior of the PEMFC.

**Figure 5:**
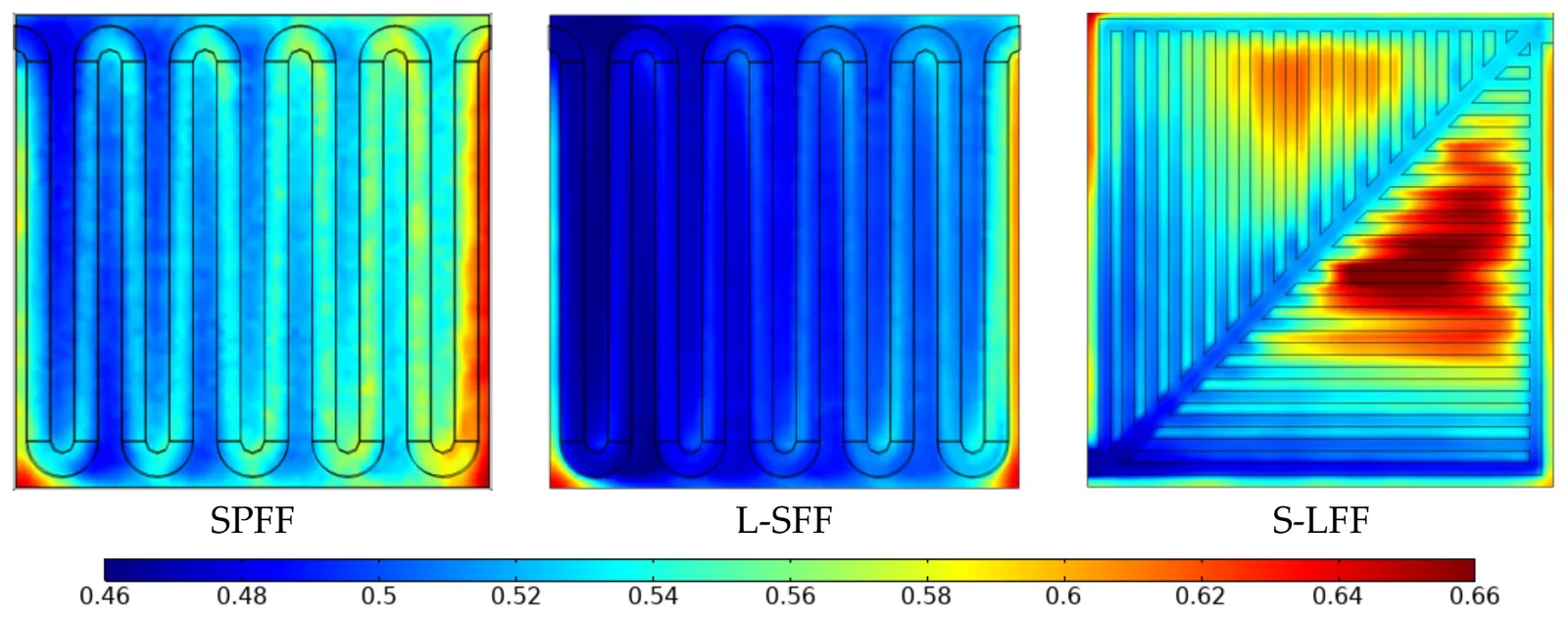
Distribution of the molar fraction of water at the cathode.

Figure 5 presents the membrane water content distribution under an output voltage of 0.6 V for different flow field configurations. The results show that the L-SFF flow field achieves an average water mole fraction of 0.513, with a lower maximum water concentration compared with other configurations. This indicates that the composite flow field contributes to a more uniform membrane hydration distribution and reduces localized water accumulation. In comparison, the SPFF flow field exhibits relatively uniform water distribution with an average mole fraction of 0.566, demonstrating its capability in maintaining a balanced water distribution. The lower water concentration near the outlet region suggests that the serpentine structure provides favorable transport pathways for water redistribution.

The S-LFF flow field also shows relatively uniform water distribution; however, higher water concentrations remain near the lower outlet region, indicating less uniform local water distribution. Overall, comparative analysis demonstrates that the L-SFF flow field provides improved membrane water distribution and water management characteristics. The combination of leaf-vein branching and serpentine channels enables coordinated reactant transport and water distribution, contributing to a more stable electrochemical reaction environment.

#### 3.1.3. Analysis of the Hydrogen Distribution Pattern at the Anode

**Figure 6:**
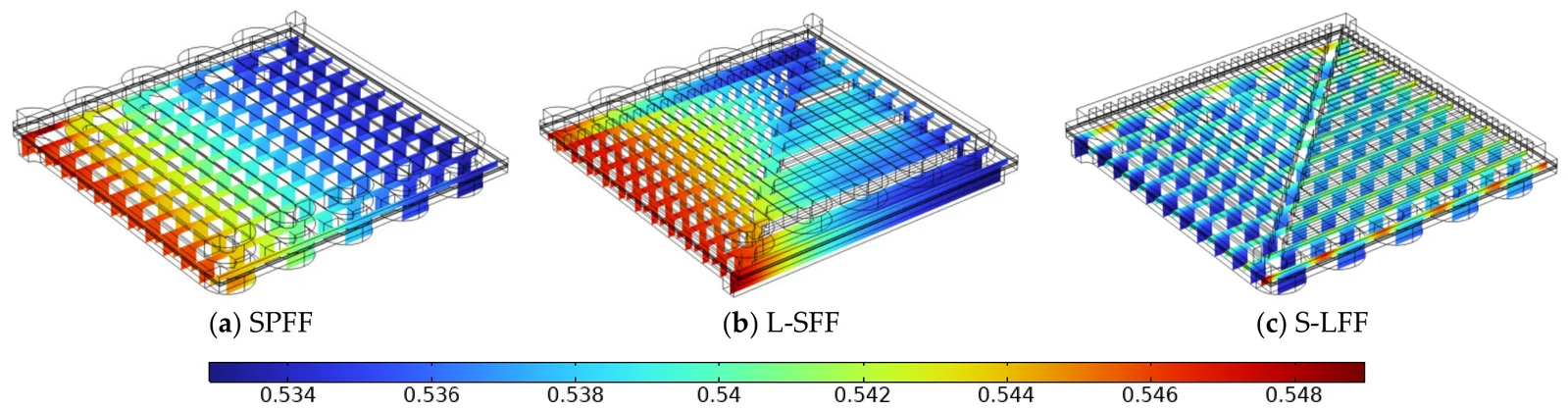
Hydrogen molar fraction distribution within the flow channels at an output voltage of 0.6 V.

Figure 6 illustrates the hydrogen distribution at the anode of the PEMFC under an operating temperature of 353 K and an output voltage of 0.6 V for different flow field configurations. Numerical simulations of the three flow fields yielded average hydrogen mole fractions of approximately 0.545 for SPFF, 0.552 for L-SFF, and 0.536 for S-LFF.

The results indicate that hydrogen flows uniformly along the main channels throughout the anode, with the primary trunks achieving high transport efficiency. The L-SFF maintains a macroscopically uniform hydrogen concentration, preventing localized low-flow regions and ensuring adequate reactant supply across the entire active area. Local analysis reveals slightly higher hydrogen concentrations at the junctions of the main trunks and branches, while the terminal branch regions exhibit slightly lower concentrations; however, the reduction is minimal. This demonstrates that the leaf-vein branching network effectively coordinates multiple transport pathways, mitigating the reactant depletion commonly observed at the ends of conventional single-channel flow fields and improving overall reaction efficiency in the anode active region.

Comparison across flow field structures shows that pure serpentine channels exhibit high hydrogen concentrations near the inlet but experience significant depletion near the outlet, which may lead to non-uniform reactions and performance degradation. In contrast, SPFF and F-SFF flow fields, due to efficient water removal and optimized branching, achieve more uniform membrane water distribution, with moderate average mole fractions and only minor water accumulation near the membrane outlet, effectively reducing flooding risk. The L-SFF flow field exhibits the lowest average water mole fraction and a more balanced water distribution while maintaining efficient gas transport and micro-scale drainage. Overall, these analyses indicate that the L-SFF configuration performs best in minimizing membrane flooding risk, enhancing water thermal management, and maintaining uniform hydrogen distribution, thereby significantly improving PEMFC durability and overall operational efficiency.

#### 3.1.4. Analysis of PEM Temperature Distribution

**Figure 7:**
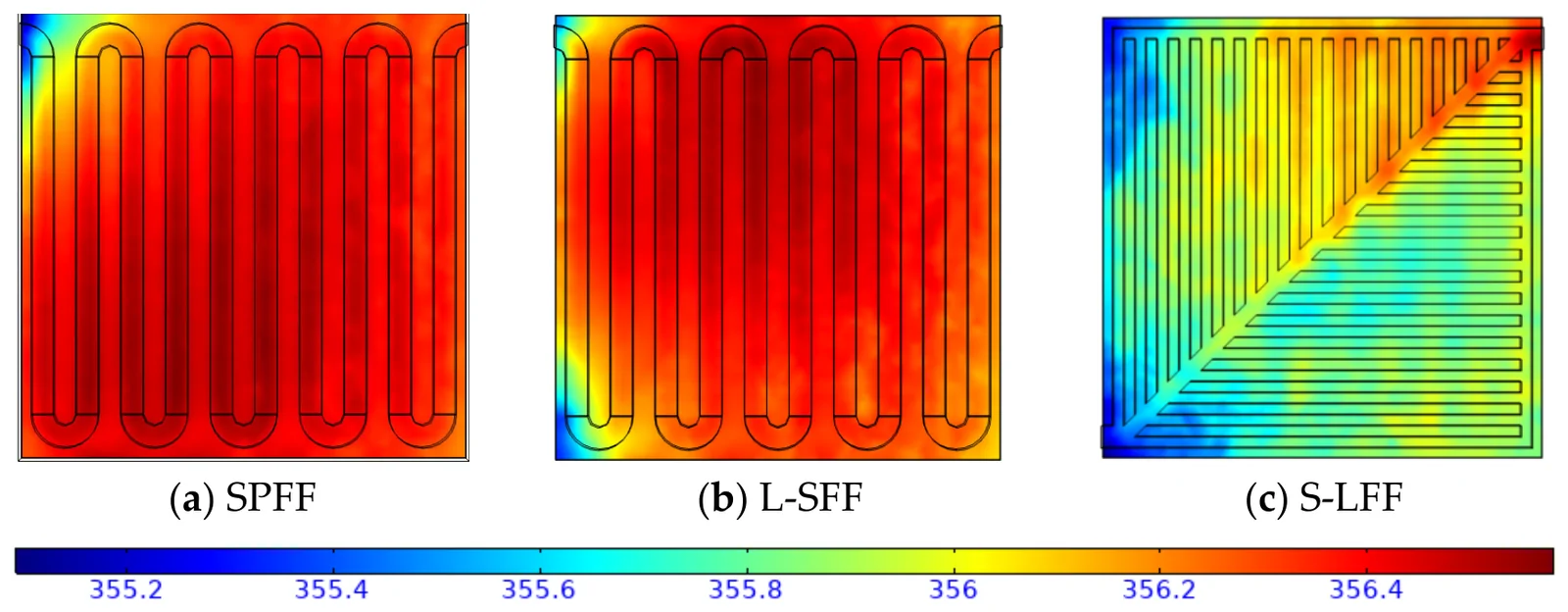
Temperature distribution map of the membrane.

Figure 7 illustrates the spatial temperature distribution of the proton exchange membrane under different flow field configurations. The simulation results reveal differences in both average temperature and temperature uniformity among the investigated designs. For the SPFF flow field, the membrane exhibits an average temperature of 356.16 K, with a maximum temperature of 356.64 K and a temperature standard deviation of approximately 0.18 K. The elevated temperature regions are mainly concentrated near the outlet, indicating localized thermal accumulation caused by non-uniform reactant transport.

For the L-SFF flow field, the average membrane temperature is slightly higher at 356.61 K, while the maximum temperature decreases slightly to 356.58 K. Moreover, the temperature standard deviation is reduced to approximately 0.14 K, indicating a more uniform thermal distribution compared with SPFF. This improvement is attributed to the coordinated transport characteristics of the main trunk and multi-level branching network, which promote a more uniform reactant supply and reaction distribution, thereby reducing local temperature gradients and hotspot intensity. Therefore, the thermal advantage of L-SFF is mainly reflected in improved temperature uniformity rather than simply lower average temperature.

Although the S-LFF flow field presents a lower average temperature of 355.89 K, its temperature uniformity is not superior to that of L-SFF. The remaining localized high-temperature regions near the outlet and less effective reaction distribution result in a temperature standard deviation of approximately 0.16 K. Meanwhile, the lower electrochemical performance of S-LFF indicates that the reduced average temperature does not necessarily correspond to improved overall thermal management.

Comparative analysis demonstrates that L-SFF achieves the best balance between thermal uniformity and electrochemical performance among the investigated configurations. The leaf-vein–serpentine composite structure effectively reduces local temperature fluctuations while maintaining enhanced reactant transport and reaction uniformity. These results indicate that L-SFF provides a more favorable thermal environment for PEMFC operation compared with conventional and single-structure biomimetic flow fields.

#### 3.1.5. Distribution of Membrane Current Density

**Figure 8:**
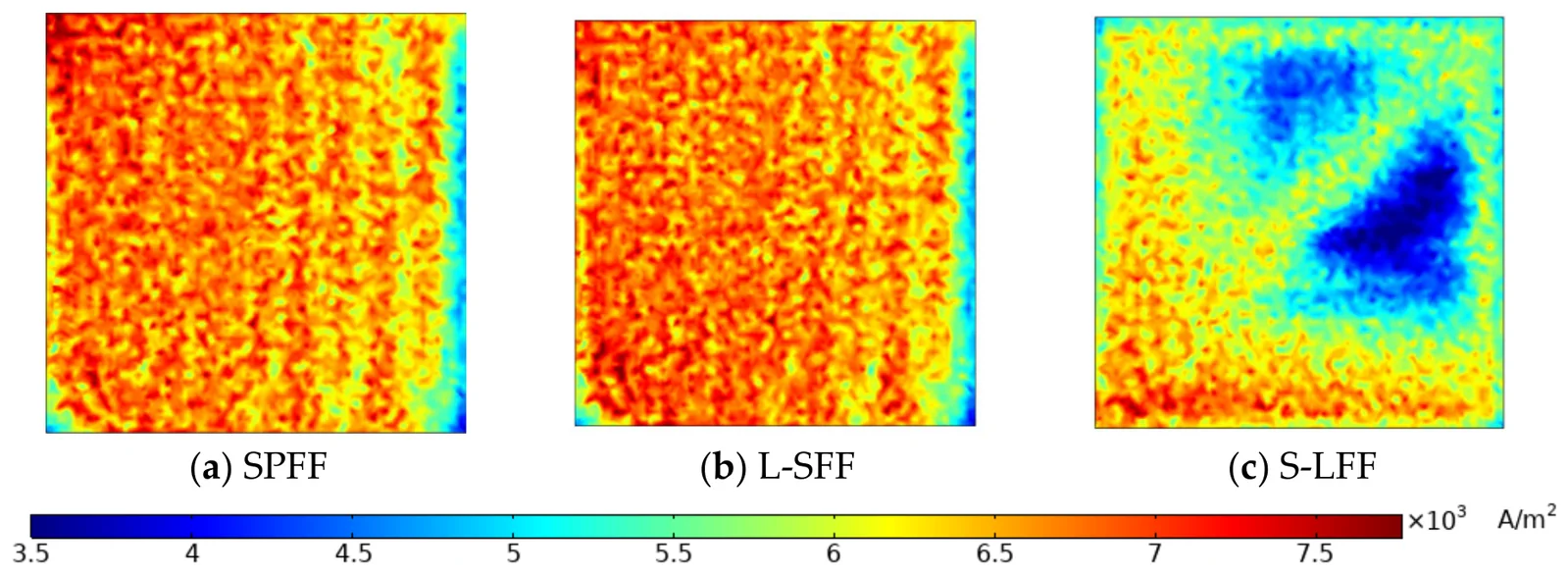
Membrane current density distribution for different flow channels at an output voltage of 0.6 V.

Under an output voltage of 0.6 V, the membrane current density distributions for the three flow field configurations were analyzed, as shown in Figure 8. The SPFF flow field exhibits the highest average membrane current density at 7239.6 A/m^2^, with a uniformity index of 0.113, indicating relatively even reaction across the membrane surface and minimal low-flow regions. The L-SFF flow field has a slightly lower average current density of 7092.0 A/m^2^ but a uniformity index of only 0.135, suggesting more balanced local distributions with reduced fluctuations. In contrast, the S-LFF flow field shows a significantly lower average current density of 5622.1 A/m^2^ and a high uniformity index of 0.273, with pronounced local variations and two low-current-density regions near the outlet, reflecting polarization effects that may damage the membrane and reduce fuel cell economic performance.

Overall, the L-SFF flow field achieves a favorable balance of high average current density and improved uniformity, benefiting from the synergistic effect of the leaf-vein branching network and the serpentine main trunk. The trunk channels rapidly deliver reactant gases to the branches, while the branches effectively mitigate localized high flow and water concentration zones, resulting in a more uniform current density distribution across the active membrane area. Compared with the single serpentine and S-LFF configurations, the L-SFF flow field not only optimizes overall electrochemical reaction efficiency but also minimizes over- and under-flow regions, enhancing operational stability and durability at high power densities. Therefore, the L-SFF design demonstrates clear superiority in balancing average performance and uniformity, providing a reliable reference for high-performance PEMFC design.

#### 3.1.6. Analysis of Voltage Drop Across the Three Flow Channels for the Anode and Cathode

Pressure loss is an important parameter for evaluating flow-field performance, as excessive pressure drop increases parasitic energy consumption and reduces the overall efficiency of PEMFC systems. Therefore, the pressure drops on both the anode and cathode sides of SPFF, S-LFF, and L-SFF were quantitatively compared.

**Figure 9:**
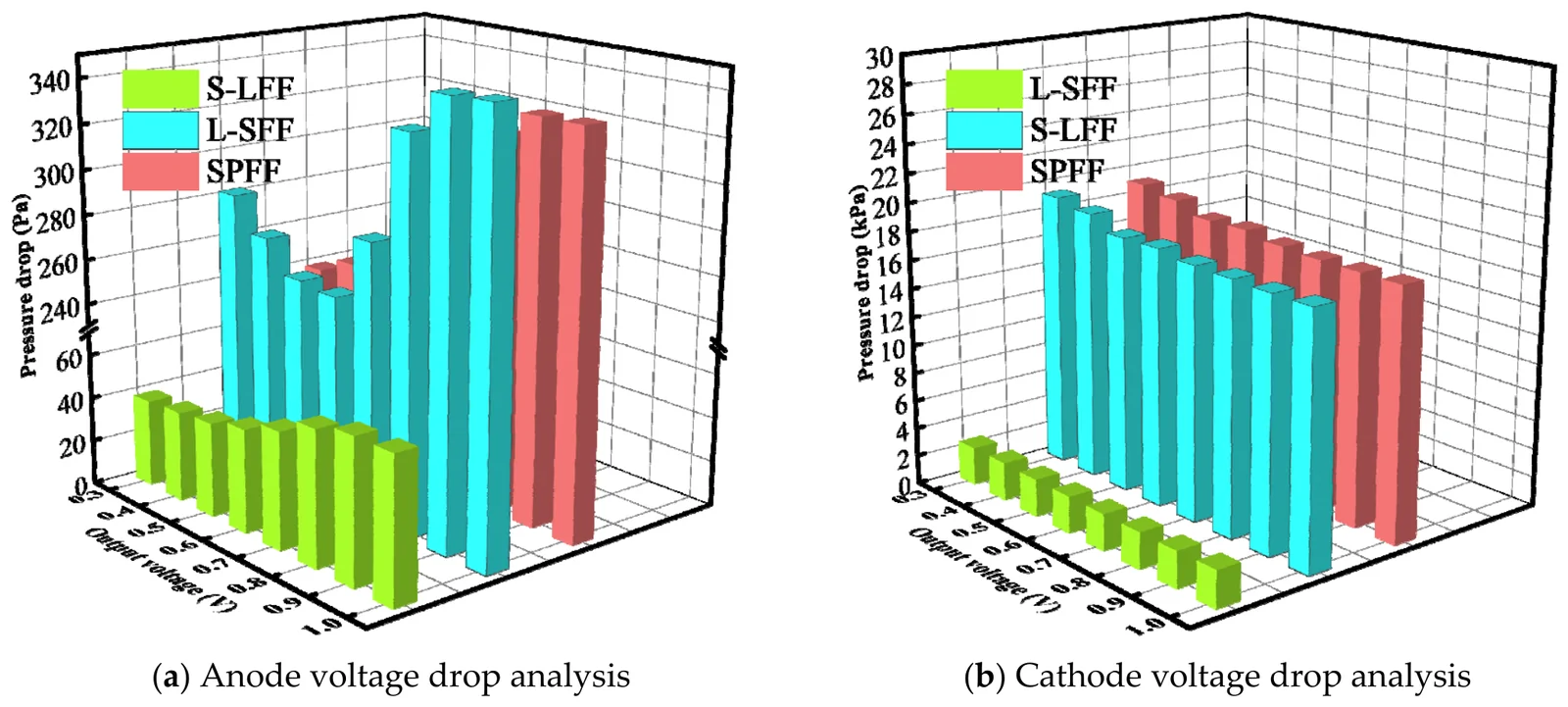
Three types of flow channel anode-cathode voltage drop distribution diagrams.

As shown in Figure 9a, the anode pressure drops of SPFF and L-SFF are 242–340 Pa and 251–349 Pa, respectively, showing similar flow resistance characteristics. This is mainly because both configurations retain the serpentine main-channel structure, where the long flow path and repeated bends generate considerable flow resistance. For L-SFF, although the leaf-vein branching network improves gas distribution, the additional branch junctions introduce local flow resistance, resulting in a pressure drop comparable to that of SPFF. In contrast, S-LFF exhibits a lower anode pressure drop (38.9–64 Pa), which is attributed to the multi-branch parallel pathways of the leaf-vein structure that reduce local velocity and flow resistance.

As shown in Figure 9b, the cathode pressure drop is generally higher than that on the anode side, mainly due to the higher air supply requirement for oxygen transport and the additional resistance caused by water generation during the cathodic reaction. The cathode pressure drops of SPFF and S-LFF range from 17.45 to 19.30 kPa, mainly due to the higher flow resistance associated with continuous serpentine channels. In comparison, L-SFF exhibits a lower cathode pressure drop of 2.51–2.65 kPa. This reduction is attributed to the hierarchical leaf-vein branching structure, which distributes gas flow through multiple pathways, reduces local velocity, and decreases flow resistance.

Furthermore, GDL porosity variation has a limited influence on the overall pressure loss, as all flow-field configurations maintain relatively stable pressure-drop characteristics. These results indicate that L-SFF can enhance reactant transport and water management while maintaining low flow resistance, achieving a better balance between performance improvement and auxiliary energy consumption.

### 3.2. Effect of Porosity Distribution on PEMFC Performance

**Figure 10:**
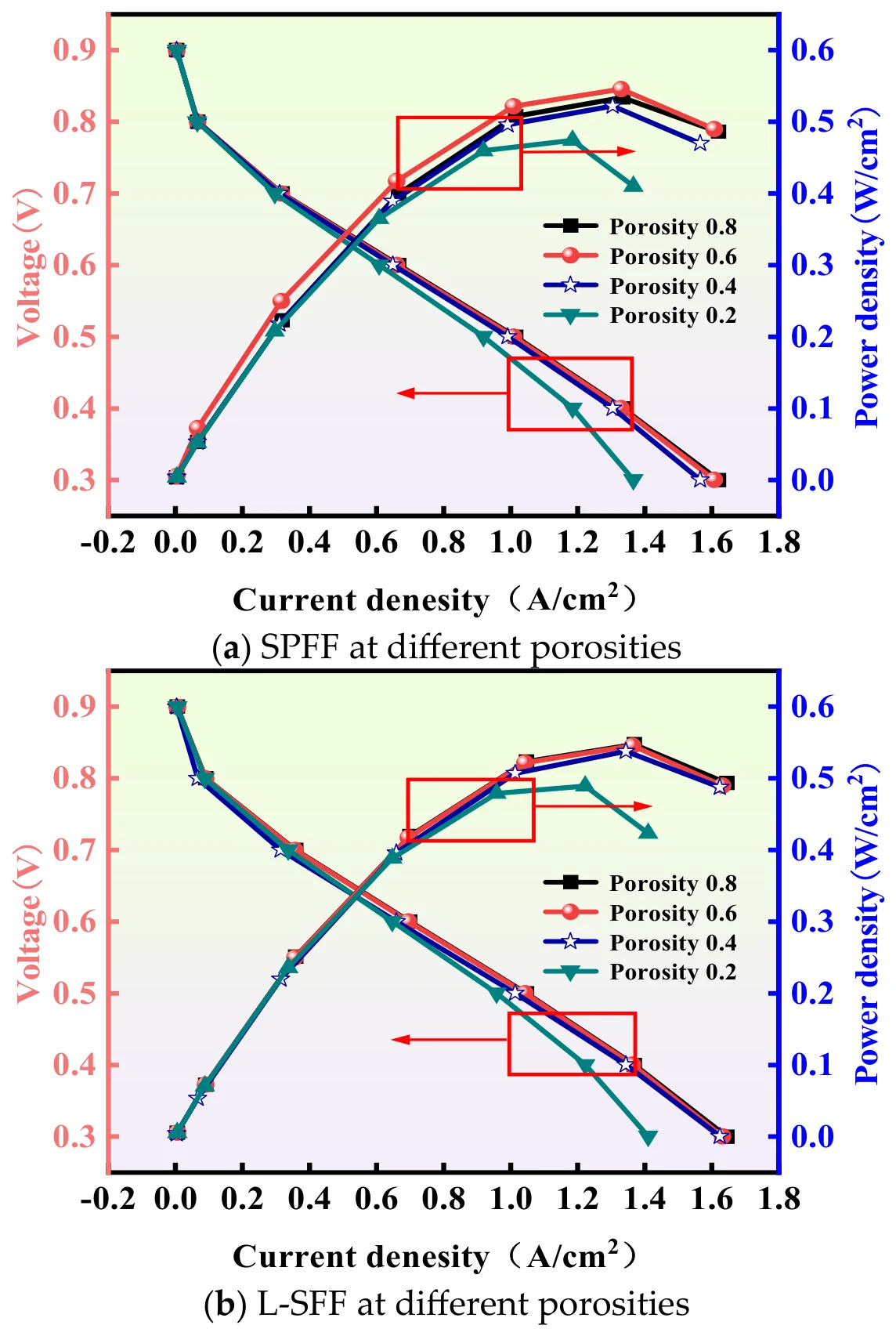
Performance analysis of SPFF and L-SFF at an output voltage of 0.6 V under different porosity conditions.

By quantitatively analyzing the polarization and power density curves of the SPFF and L-SFF flow fields under different GDL porosities of 0.2, 0.4, 0.6, and 0.8, it is found that the L-SFF structure exhibits a clear advantage in performance stability. In the simulation, GDL porosity variation was coupled with changes in porous-media transport properties. Specifically, the effective diffusivity and permeability were modified according to the porosity-dependent correlations introduced in the model section, while other material properties were assumed to remain constant. As shown in Figure 10, for the SPFF flow field, a decrease in porosity leads to a significant reduction in average voltage, with the maximum power density decreasing from approximately 0.534 W/cm^2^ to 0.474 W/cm^2^, corresponding to a reduction of 11.2%. This performance degradation is mainly attributed to restricted gas transport and increased local liquid water retention under low-porosity conditions, which result in non-uniform membrane current density distribution and insufficient local reaction efficiency.

In contrast, the L-SFF leaf-vein–serpentine composite flow field shows substantially reduced sensitivity to GDL porosity. When the porosity decreases from 0.8 to 0.2, the maximum power density only decreases from approximately 0.547 W/cm^2^ to 0.489 W/cm^2^, corresponding to a reduction of 10.6%, while the membrane current density distribution remains highly uniform. This indicates that the L-SFF flow field, through the synergistic effect of the leaf-vein branching network and the serpentine main trunk, enables balanced gas distribution within the membrane and efficient water removal, thereby effectively mitigating the adverse effects of GDL porosity variation on electrochemical performance.

Further analysis shows that the L-SFF flow field maintains high local current density even under high current density operating conditions, avoiding over- or under-flow regions and ensuring sufficient reaction across the membrane active area. This configuration not only enhances overall cell power output but also improves local thermal load distribution and water thermal management, resulting in more uniform membrane temperature and water content. Collectively, these results demonstrate that the leaf-vein–serpentine composite design significantly reduces the impact of GDL porosity fluctuations on fuel cell performance, creating a more stable, efficient, and uniform electrochemical environment. Therefore, the L-SFF configuration represents an effective strategy for optimizing PEMFCs for high power density and stable operation under varying GDL transport conditions.

### 3.3. Effect of Water Distribution on PEMFC Performance

**Figure 11:**
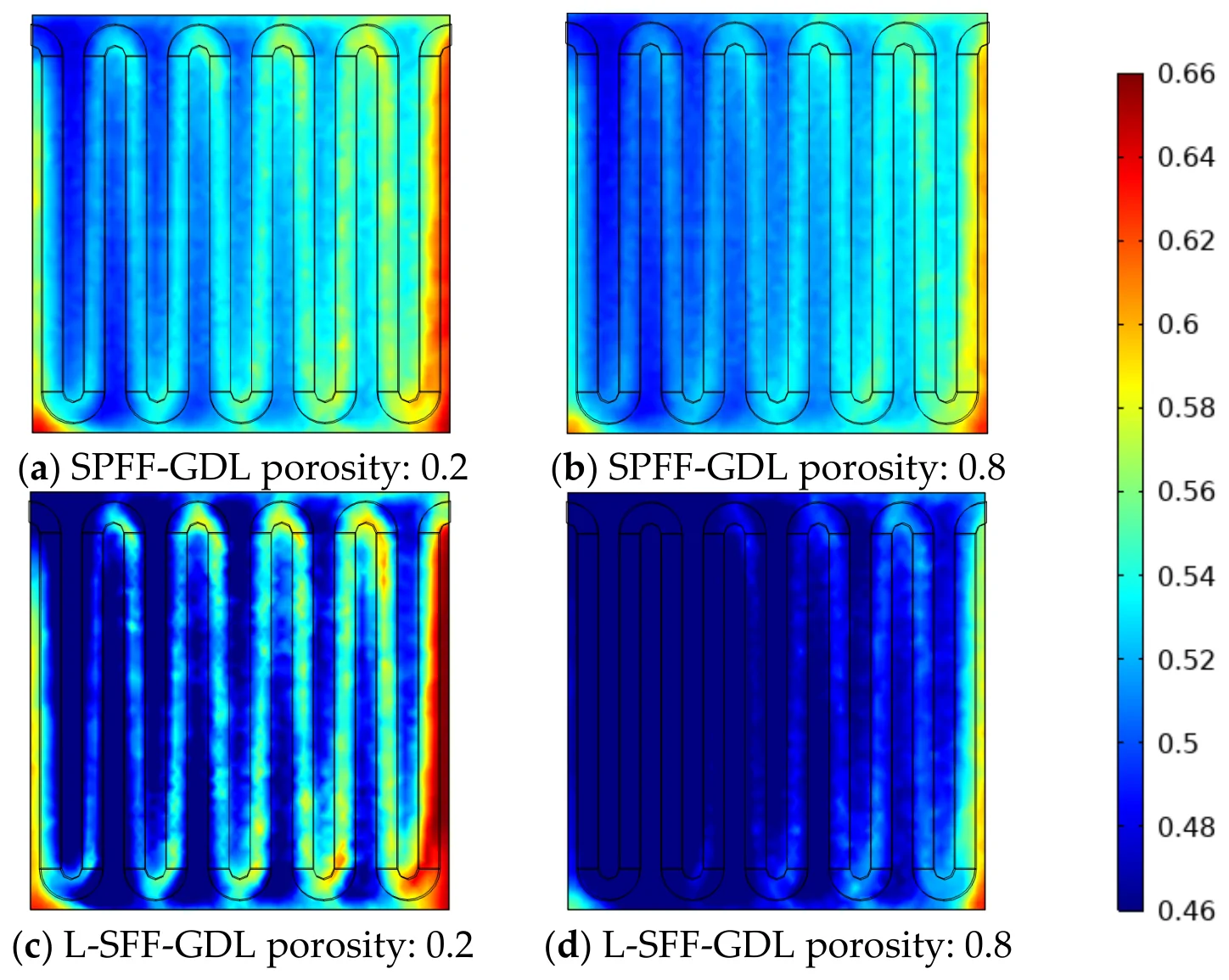
Membrane water concentration distributions at GDL porosities ranging from 0.2 to 0.8.

Figure 11 illustrates the variation in membrane water concentration distribution for SPFF and L-SFF flow field configurations when the GDL porosity changes from 0.2 to 0.8. Based on the color scale ranging from 0.46 to 0.66, the average membrane water mole fractions of SPFF at GDL porosities of 0.2 and 0.8 are estimated to be approximately 0.528 and 0.517, respectively. Under the same porosity conditions, the corresponding values for L-SFF are approximately 0.492 and 0.468. These results indicate that increasing GDL porosity reduces the membrane water content in both flow field configurations, suggesting that higher porosity facilitates gas diffusion and liquid water removal, thereby lowering the risk of water accumulation within the membrane.

For the SPFF configuration, the membrane water concentration remains relatively high at low GDL porosity, with a pronounced high-water-content region near the outlet side. This indicates that low porosity restricts water diffusion and removal from the membrane through the GDL and flow channel, thereby increasing the possibility of local flooding. When the GDL porosity increases to 0.8, the membrane water concentration decreases to some extent; however, water accumulation is still observed near the outlet. This suggests that although the conventional serpentine flow field provides certain drainage capability, its long-channel structure may still lead to local water accumulation. In contrast, the average membrane water mole fraction of L-SFF is reduced by approximately 6.8% compared with SPFF at a porosity of 0.2 and by approximately 9.3% at a porosity of 0.8, demonstrating that the leaf-vein–serpentine composite structure can effectively suppress water accumulation within the membrane.

From the perspective of spatial distribution, the L-SFF configuration exhibits a lower and more concentrated water content level under high-GDL-porosity conditions. Most regions of the membrane remain within the low-water-concentration range, with only a slight increase near the outlet. This indicates that the synergistic effect of the leaf-vein branching network and the serpentine main channel improves both reactant transport and water removal pathways, allowing generated water to be more efficiently discharged through the GDL into the flow channel. Overall, increasing GDL porosity improves membrane water management, while the L-SFF composite flow field further enhances this effect, enabling the PEMFC to maintain better water control capability and a more stable electrochemical reaction environment under different porosity conditions.

### 3.4. Membrane Current Density Distribution

**Figure 12:**
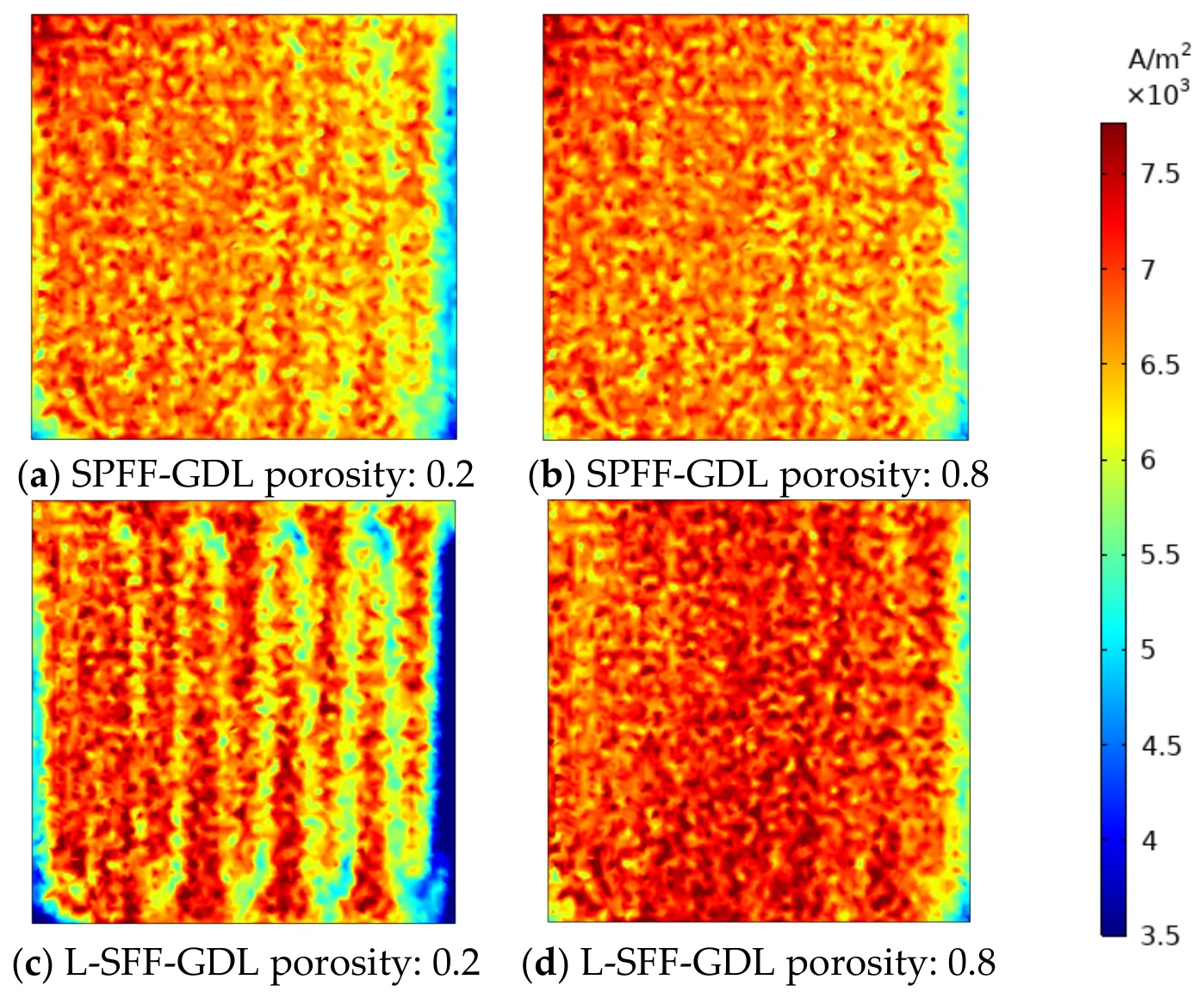
Membrane current density distributions at GDL porosities ranging from 0.2 to 0.8.

A comparative analysis of membrane current density distributions under different GDL porosities is conducted for the SPFF and L-SFF configurations, as shown in Figure 12. Overall, the GDL porosity has a significant influence on the spatial distribution of membrane current density. For the SPFF flow field, when the GDL porosity is 0.2, the maximum and minimum membrane current densities are 8.65 × 10^3^ A/m^2^ and 3.53 × 10^3^ A/m^2^, respectively. When the porosity increases to 0.8, the maximum current density increases to 8.79 × 10^3^ A/m^2^, while the minimum value rises to 4.16 × 10^3^ A/m^2^. This indicates that a higher GDL porosity improves reactant gas diffusion within the GDL, alleviates local mass transport limitations, and reduces low-current-density regions, thereby promoting more sufficient electrochemical reactions over the membrane surface.

For the L-SFF leaf-vein–serpentine composite flow field, when the GDL porosity is 0.2, the maximum membrane current density reaches 8.58 × 10^3^ A/m^2^, whereas the minimum value is only 2.10 × 10^3^ A/m^2^. This suggests that, under low-porosity conditions, gas diffusion and water removal through the GDL are strongly restricted, resulting in local reactant starvation or water retention and consequently expanding low-current-density regions. When the porosity increases to 0.8, the maximum current density of the L-SFF flow field reaches 8.77 × 10^3^ A/m^2^, and the minimum value increases to 3.92 × 10^3^ A/m^2^. The low-current-density regions are significantly reduced, and the overall distribution becomes more continuous, indicating that increasing GDL porosity effectively enhances mass transport capability and local reaction uniformity under the L-SFF configuration.

Comparing the two flow fields, under high-GDL-porosity conditions, the maximum current densities of SPFF and L-SFF are very close, at 8.79 × 10^3^ A/m^2^ and 8.77 × 10^3^ A/m^2^, respectively, indicating that both configurations can achieve high local reaction intensity when gas transport resistance is reduced. However, from the spatial distribution perspective, the L-SFF configuration maintains better continuity of current density at high porosity, demonstrating that the leaf-vein branching network and serpentine main channel synergistically improve reactant transport pathways and promote more sufficient gas diffusion toward the membrane electrode active area. Overall, increasing GDL porosity helps raise the lower limit of membrane current density and mitigate local mass transport limitations, while the L-SFF composite flow field can better exploit its branching-guidance and water-removal advantages under high-porosity conditions, providing a more stable and uniform electrochemical reaction environment for PEMFC operation.

## 4. Conclusions

In this study, a leaf-vein–serpentine composite flow field (L-SFF) was proposed to improve the electrochemical performance, gas distribution uniformity, and water–thermal management of PEMFCs. A three-dimensional multiphysics model was established in COMSOL Multiphysics 6.1 to compare the traditional serpentine flow field (SPFF), cathode leaf-vein–serpentine flow field (S-LFF), and anode leaf-vein–serpentine flow field (L-SFF). The influence of GDL porosity on cell performance was also investigated. The main conclusions are as follows:

1. Compared with the traditional SPFF structure, the L-SFF design improves PEMFC output performance. The L-SFF adopts a main trunk and multi-level branch structure inspired by coconut palm leaf veins, with a branch angle of 45° and a branch width of 2 mm. Under 0.6 V, 353 K, and 1 bar operating conditions, the maximum power density of L-SFF reaches 0.538 W/cm^2^, which is approximately 2.1% higher than that of SPFF. This indicates that arranging the leaf-vein structure on the anode side can enhance hydrogen transport and improve overall electrochemical reaction efficiency.
2. The L-SFF flow field shows improved gas distribution and water management characteristics. The average anode current density reaches 7092.0 A m^−2^, while the membrane water mole fraction is 0.513, indicating a more uniform membrane hydration state. The current density uniformity index is approximately 0.135, demonstrating that L-SFF maintains a relatively uniform reaction distribution and reduces severe local over-reaction or under-reaction regions. Meanwhile, the average membrane temperature is approximately 356.61 K, with weakened local hotspots and reduced temperature gradients. These characteristics contribute to a more uniform operating environment and improved operational stability of the PEMFC.
3. The L-SFF design reduces the sensitivity of PEMFC performance to GDL porosity variations. When GDL porosity decreases from 0.8 to 0.2, the maximum power density of SPFF decreases by 11.2%, whereas that of L-SFF decreases by only 10.6%. This demonstrates that the leaf-vein–serpentine structure can alleviate mass transfer limitations caused by low porosity and maintain a more stable electrochemical environment.

Overall, the L-SFF flow field effectively improves power output, reactant distribution, water management, and thermal uniformity. By reducing local transport non-uniformity and providing a more stable operating environment, this design offers a potential strategy for PEMFC flow-field optimization, while further durability evaluation requires degradation modeling and experimental validation.

## Figures and Tables

**Table 1:** Geometry parameters of the model.

Parameter	Value	Unit
Channel length	20	mm
Channel height	1	mm
Thickness (GDL, CL, PEM)	0.4, 0.05, 0.1	mm
BP width	2	mm
Effective area	400	mm^2^

**Table 2:** Operating conditions and physical parameters.

Parameter	Value	Unit
**Operating conditions**		
Operation temperature	353.15	K
Operation pressure	101,325	Pa
Reference concentration (Hydrogen, oxygen)	40.88, 40.88	mol·m^−3^
**Physical parameters**		
Electrical conductivity (BP, GDL, CL)	83,000, 1000, 1000	S·m^−1^
Intrinsic permeability (GDL, CL)	1.18 × 10^−11^, 2.36 × 10^−10^	m^2^
Thermal conductivity (BP, GDL, CL, PEM)	85.5, 1.7, 8,2	W·m^−1^·K^−1^
Porosity (GDL, CL)	0.5, 0.3	—
Dry PEM density	1980	kg·m^3^
Reference exchange current density (Cathode, anode)	0.001, 100	A·m^−2^

## Data Availability

The data presented in this study are not publicly available due to confidentiality restrictions.
